# Physician–Pharmacist Collaborative Clinic Model to Improve Anticoagulation Quality in Atrial Fibrillation Patients Receiving Warfarin: An Analysis of Time in Therapeutic Range and a Nomogram Development

**DOI:** 10.3389/fphar.2021.673302

**Published:** 2021-06-09

**Authors:** Na Wang, Sha Qiu, Ya Yang, Chi Zhang, Zhi-Chun Gu, Yan Qian

**Affiliations:** ^1^ Department of Pharmacy, The Second Affiliated Hospital of Chongqing Medical University, Chongqing, China; ^2^ Department of Infection Control, Renji Hospital, School of Medicine, Shanghai Jiaotong University, Shanghai, China; ^3^ Department of Pharmacy, Renji Hospital, School of Medicine, Shanghai Jiaotong University, Shanghai, China; ^4^ Shanghai Anticoagulation Pharmacist Alliance, Shanghai Pharmaceutical Association, Shanghai, China; ^5^ Chinese Society of Cardiothoracic and Vascular Anesthesiology, Beijing, China

**Keywords:** atrial fibrillation, anticoagulation, warfarin, time in therapeutic range, prediction model, clinical pharmacist

## Abstract

**Background:** Poor time in therapeutic range (TTR) control is associated with an increased risk of stroke and bleeding in atrial fibrillation (AF) patients receiving warfarin. This study aimed to determine whether the physician–pharmacist collaborative clinic (PPCC) model could improve the anticoagulation quality as well as to create a nomogram for predicting anticoagulation quality in AF patients.

**Methods:** This retrospective observational study enrolled AF patients who either initially received warfarin or returned to warfarin after withdrawal between January 1, 2016 and January 1, 2021, at our institution. The primary outcome was dynamic changes in TTRs (a TTR of ≥60% considered high anticoagulation quality). The secondary outcomes were thromboembolic and bleeding events during follow-up. We compared the dynamic changes in TTRs between the general clinic (GC) and PPCC groups in both the original and propensity score matching (PSM) cohorts. In addition, we explored the potential predictors of high anticoagulation quality and subsequently formulated a nomogram to predict anticoagulation quality.

**Results:** A total of 265 patients with AF were included, comprising 57 patients in the PPCC group and 208 patients in the GC group. During a median follow-up period of 203 days, the PPCC group had a shorter time (76 vs. 199 days, *p* < 0.001) and more patients achieved a TTR ≥60% (73.7 vs. 47.1%, *p* = 0.002 by log-rank test) than the GC group. The results from the PSM cohort confirmed this finding. No significant differences in the incidences of thromboembolic events (5.3 vs. 5.3%, *p* = 1.000) and bleeding events (4.3 vs. 3.5%, *p* = 1.000) were observed between the two groups. Four variables were explored as predictors related to high anticoagulation quality: treatment within a PPCC, history of bleeding, history of bleeding, and the presence of more than four comorbidities. The nomogram revealed a moderate predictive ability (c-index: 0.718, 95% confidence interval (95%CI): 0.669–0.767) and a moderately fitted calibration curve.

**Conclusion:** The PPCC model contributed to improved anticoagulation quality in AF patients receiving warfarin. The nomogram might be an effective tool to predict anticoagulation quality and could aid physicians and pharmacists in the selection of patients who will likely benefit from sustained and active intervention.

## Introduction

The incidence of atrial fibrillation (AF), the most common sustained arrhythmia (Ball et al., 2013), has increased by 33% during the past 20 years, resulting in 37,574 million cases worldwide by 2017 (Lippi et al., 2020). Of importance, ischemic stroke is a major complication related to AF (Chien et al., 2010), which accounted for the third highest burden of cardiovascular disease with a disease burden of 55.1 million disability-adjusted life years based on a global survey in 2017 (DALYs and Collaborators, 2018). Anticoagulation therapy is key to prevent AF-associated stroke (Lip et al., 2017). Although non-vitamin K oral anticoagulants (NOACs) are now recommended as a priority in non-valvular AF patients, both by the European Society of Cardiology (ESC) (Hindricks et al., 2020) and the American Heart Association (AHA) (January et al., 2019), warfarin still plays an important role in anticoagulation therapy for AF patients because of its wider scope of application (mechanical valves, moderate to severe mitral stenosis, etc.) and very low cost. To ensure the effectiveness and safety of warfarin in clinical practice, it is necessary to frequently monitor the international normalized ratio (INR) and to adjust the dosage accordingly (Gu et al., 2019). Time in therapeutic range (TTR), which refers to the percentage of time when the INR values remain within the therapeutic range, is commonly applied as a measure of the anticoagulation quality of warfarin therapy within a given time frame because of its association with the occurrence of bleeding and thromboembolic events (Schmitt et al., 2003). Given that warfarin has a narrow therapeutic window and its anticoagulant effect is susceptible to numerous factors (such as diet, drugs, and gene polymorphisms) (Gu et al., 2018), tailoring warfarin treatment to the case at hand is a challenge for both patients and physicians. Clinical pharmacists, as the main providers of pharmaceutical professional services, can provide good anticoagulant pharmaceutical services to physicians and patients in the formulation of medication regimens and provision of medication education. Previous studies have shown that the participation of clinical pharmacists could improve the TTR in AF patients receiving warfarin (An et al., 2017; Kose et al., 2018; Marcatto et al., 2018). However, these studies only focused on the relationship between pharmacists’ intervention and outcomes at a certain time point and did not explore the effect of the continuous intervention on the dynamic changes of TTRs. In addition, some studies have explored the risk factors associated with the anticoagulation quality of warfarin (Okumura et al., 2011; Pokorney et al., 2015; Mwita et al., 2018), but the results have not been extrapolated to clinical practice based on score systems. Hence, this study aimed to investigate the role of physician–pharmacist collaborative clinic (PPCC) model on anticoagulation quality as well as to create a clinical prediction model for anticoagulation quality in AF patients receiving warfarin.

## Methods

### Study Design and Participants

Between January 1, 2016 and January 1, 2021, we conducted a retrospective observational study in a cohort of AF patients who received warfarin at our institution (Ethics Registration: NO. 2020-411). The inclusion criteria were as follows: 1) had been diagnosed with AF confirmed by 12 lead- electrocardiography (ECG), pacemaker/implantable cardioverter defibrillator electrocardiogram findings, or Holter ECG (Hindricks et al., 2020); 2) aged ≥18 years; 3) were new users of warfarin or users who resumed treatment after discontinuing warfarin for ≥12 months; 4) had been taking warfarin for at least 6 weeks; 5) had at least three eligible INR values, of which the interval between any two adjacent INR measurements was ≤9 weeks. The exclusion criteria were as follows: 1) had a prescription filled for warfarin during the 12 months prior to the initiation of warfarin treatment and 2) had <90 evaluable days or missing baseline data. The index date was set at 7 weeks after the first claim of warfarin prescription. According to the site where the patient visited our institution, patients were divided into a PPCC group and a general clinic (GC) group.

### Management Model of PPCCs in the Care of AF Patients

In this study, the clinical pharmacists (N.W., S.Q., C.Z., and Z.G.) in PPCCs had received standardized training and obtained corresponding certificates. In brief, when a patient visited the PPCC, a diagnosis was made according to relevant physical examinations by the physicians. Clinical pharmacists then conducted an investigation to collect relative information, including demographic characteristics, comorbidities, laboratory parameters, co-administered drugs and foods, adverse reactions, thromboembolism events, bleeding events, and financial situation. Based on patients’ characteristics, CHA_2_DS_2_-VASc score (congestive heart failure, hypertension, age ≥75 [doubled]; diabetes mellitus, prior stroke or transient ischemic attack [doubled], vascular disease, age 65–74 years, female) and HAS-BLED score (hypertension, abnormal renal/liver function, stroke, bleeding history or predisposition, labile international normalized ratio, elderly, drugs/alcohol) were calculated to assess stroke risk and bleeding risk, respectively (Hindricks et al., 2020). Subsequently, physicians and pharmacists jointly determined the therapeutic scheme (warfarin or non-vitamin K antagonist oral anticoagulants), treatment goal (INR target), drug dosage, treatment course, and date for the next visit. Finally, pharmacists provided standard and detailed medication education for the patient. During follow-up, pharmacists in PPCCs dynamically assessed the INR and TTR and correspondingly adjusted warfarin dose. Certainly, medication education was conducted repeatedly to improve medication adherence.

### Dynamic Assessment of Anticoagulation Quality in AF Patients

In this study, TTR is a measure of the anticoagulation quality of warfarin, which is calculated using the Rosendaal method of linear interpolation (Rosendaal et al., 1993). Specifically, this method assumes a linear relationship between two consecutive INR values, assigning a specific INR value to each patient daily. After interpolation, TTR is calculated as the percentage of time of dynamic changes during which the interpolated INR value remains within the therapeutic range. According to the antithrombotic guideline in AF (Hindricks et al., 2020), we set the therapeutic range of INR at 2.0–3.0 and considered good anticoagulation quality of warfarin when a TTR was ≥60% (Hong et al., 2017). To reflect the dynamic changes in anticoagulation quality, we dynamically calculated the TTR during the follow-up period.

### Outcome Measures and Follow-Up

The primary outcomes were dynamic changes in TTRs over time, and the secondary outcomes were clinical adverse events involving thromboembolic and bleeding events. Thromboembolic events included ischemic stroke, transient ischemic attack (TIA), and systemic embolism (SE). Bleeding events included major and minor bleeding as defined by the criteria of the International Society on Thrombosis and Haemostasis (Schulman et al., 2010). Patients in the PPCC group were followed up for at least 3 months until either a prescription was filled for a different anticoagulant or a temporary interruption in warfarin treatment due to the presence of bleeding, surgery, or other invasive procedures. The date of the final follow-up was January 1, 2021. Data in the GC group were retrospectively collected with standardized data collection forms from the electronic medical record system. Two researchers (N.W. and S.Q.) independently reviewed the forms to double check the data collected.

### Statistical Analyses

Categorical data are expressed as numbers and percentages and were compared using Chi-square or Fisher exact tests, and continuous data are reported as mean ± standard deviation and were compared using either the unpaired Student’s t-test or analysis of variance tests. To compare the dynamic changes in TTRs between the PPCC and GC groups, survival curves were generated using the Kaplan-Meier method and compared using the log-rank test. A Cox proportional hazards regression model was used to estimate hazard ratios (HRs) and 95% confidence intervals (CIs) with adjustment for potential confounders for anticoagulation quality (TTR ≥60%). The proportional hazards assumption was checked using statistical tests and graphical diagnostics based on the Schoenfeld residuals. Multivariable Cox regression model was used to determine the independent factors for anticoagulation quality. In the Cox model, time was defined as the number of days from enrollment to TTR ≥60%. Two criteria were considered necessary for a variable to be included in the Cox model: 1) a univariate *p* value indicative of anticoagulation quality ≤0.10 and 2) a plausible association with anticoagulation quality based on the data provided by our previous study (Qiu et al., 2020) or the existing literature (Moons et al., 2015). A nomogram was formulated based on the results of the multivariate Cox regression model, and the performance was assessed by the discrimination (model’s ability to distinguish between patients who did and did not achieve a TTR ≥60%, as indicated by c-index) and calibration (agreement between observed and predicted proportions of patients with TTR ≥60%, using calibration plots). C-indices were obtained with 1,000 bootstrap samples. To mitigate confounding bias between the PPCC and GC groups, propensity score matching (PSM) was performed with a 1:1 nearest matching algorithm without replacement. The balance of matched groups was assessed by standardized mean differences (SMD), and their absolute values <0.2 were considered acceptably balanced. After matching, survival curves were obtained again to confirm the primacy results. All statistical analyses were performed by an independent statistician (Y. Y.) who used R software version 4.0.3., and *p* < 0.05 was considered statistically significant.

## Results

### Patient Characteristics

**FIGURE 1:**
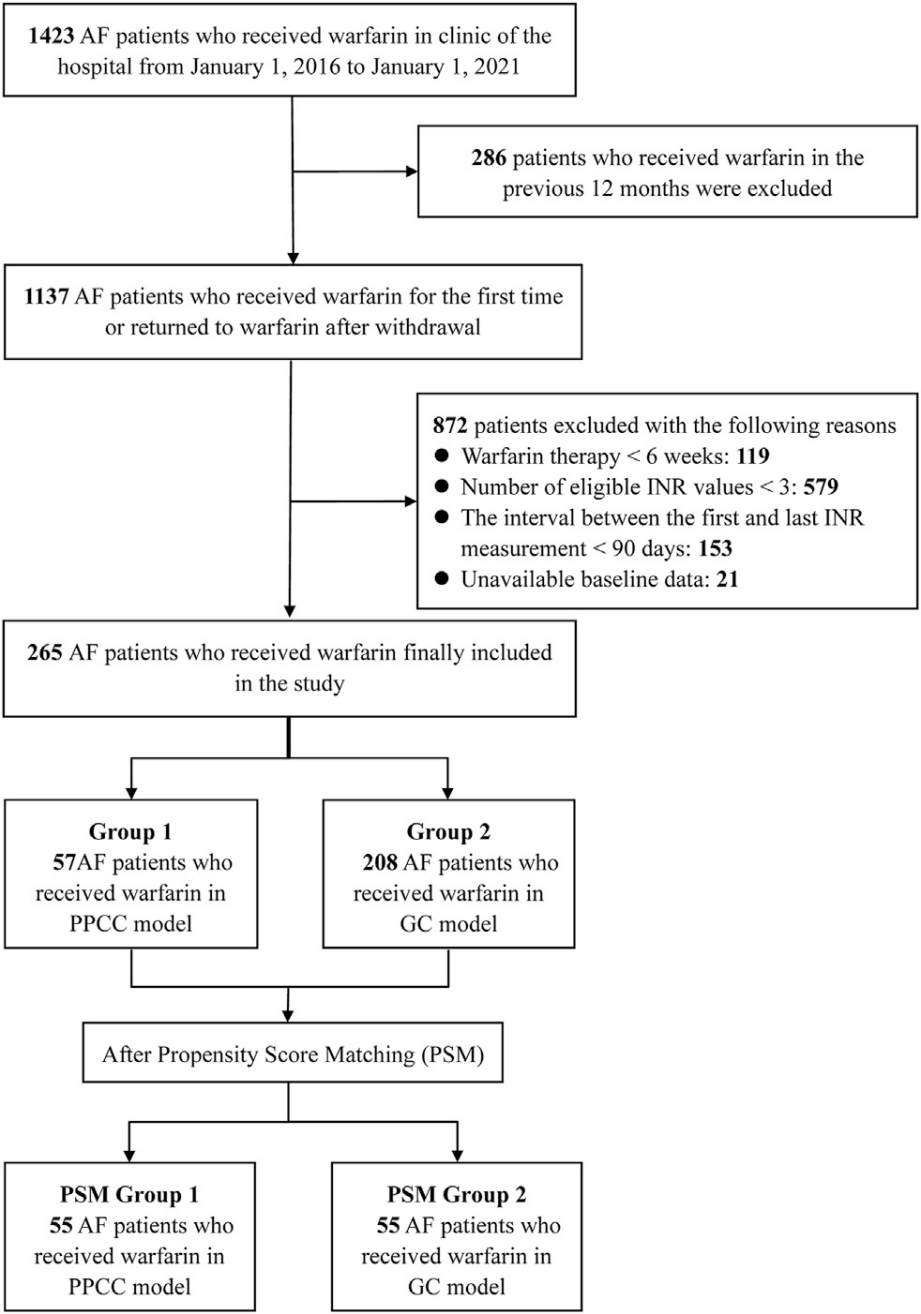
The flow diagram of selection of patients. AF, atrial fibrillation; INR, international normalized ratio; GC, general clinic; PPCC, physician–pharmacist collaborative clinic.

Between January 1, 2016 and January 1, 2021, a total of 1423 AF patients who received warfarin were initially identified, and 1,158 patients were excluded for reasons listed in Figure 1. Finally, 265 patients fulfilled the inclusion criteria, including 57 patients in the PPCC group and 208 patients in the GC group. A summary of the baseline characteristics of the included patients is presented in Table 1. The mean patient age was 69.7 ± 9.9 years, and 147 (55.5%) patients were women. Half of the patients (50.2%) had multiple cardiovascular comorbidities, including hypertension (55.5%), coronary artery disease (36.6%), and heart failure (23.8%). The baseline characteristics were relatively comparable between the PPCC and GC groups, except for age, certain comorbidities (such as deep venous thrombosis and valvular heart disease), and co-administered drugs (such as antiplatelet agents and amiodarone) (SMD >0.2 for each variable). After PSM, the baseline characteristics were well-balanced (SMD = 0.014 for age; SMD <0.001 for deep venous thrombosis, valvular heart disease, antiplatelet agents, and amiodarone), resulting in 55 patients enrolled in each group.

**TABLE 1:** Demographics and characteristics of patients before and after propensity score matching.

Variables	Original groups					Matched groups				
	Total (*n* = 265)	PPCCs (*n* = 57)	GCs (*n* = 208)	*p* Value	SMD	Total (*n* = 110)	PPCCs (*n* = 55)	GCs (*n* = 55)	*p* Value	SMD
Age, years	69.7 ± 9.9	67.1 ± 10.9	70.4 ± 9.5	0.023	0.327	68.1 ± 9.1	68.2 ± 8.8	68.0 ± 9.6	0.942	0.014
Female, *n* (%)	147 (55.5)	31 (54.4)	116 (55.8)	0.971	0.028	59 (53.6)	29 (52.7)	30 (54.5)	1.000	0.036
Comorbidities, *n* (%)										
Deep venous thrombosis	3 (1.1)	2 (3.5)	1 (0.5)	0.227	0.218	2 (1.8)	1 (1.8)	1 (1.8)	1.000	<0.001
Pulmonary embolism	1 (0.4)	0 (0.0)	1 (0.5)	1.000	0.098	0 (0.0)	0 (0.0)	0 (0.0)	NA	NA
Mechanical heart valve	9 (3.4)	2 (3.5)	7 (3.4)	1.000	0.008	4 (3.6)	2 (3.6)	2 (3.6)	1.000	0.041
Valvular heart disease	50 (18.9)	15 (26.3)	35 (16.8)	0.152	0.232	29 (26.4)	14 (25.5)	15 (27.3)	1.000	<0.001
Coronary artery disease	97 (36.6)	17 (29.8)	80 (38.5)	0.296	0.183	34 (30.9)	17 (30.9)	17 (30.9)	1.000	<0.001
Hypertension	147 (55.5)	31 (54.4)	116 (55.8)	0.971	0.028	65 (59.1)	31 (56.4)	34 (61.8)	0.698	0.111
Diabetes	38 (14.3)	9 (15.8)	29 (13.9)	0.889	0.052	17 (15.5)	9 (16.4)	8 (14.5)	1.000	0.050
Heart failure	63 (23.8)	16 (28.1)	47 (22.6)	0.494	0.126	27 (24.5)	15 (27.3)	12 (21.8)	0.658	0.127
History of stroke	36 (13.6)	5 (8.8)	31 (14.9)	0.328	0.191	9 (8.2)	5 (9.1)	4 (7.3)	1.000	0.066
History of bleeding	3 (1.1)	0 (0.0)	3 (1.4)	0.837	0.171	0 (0.0)	0 (0.0)	0 (0.0)	NA	NA
Myocardial infarction	4 (1.5)	0 (0.0)	4 (1.9)	0.659	0.198	0 (0.0)	0 (0.0)	0 (0.0)	NA	NA
≥4 Comorbidities	133 (50.2)	27 (47.4)	106 (51.0)	0.741	0.072	52 (47.3)	26 (47.3)	26 (47.3)	1.000	<0.001
Medications, n (%)										
Antiplatelet agents	33 (12.5)	4 (7.0)	29 (13.9)	0.239	0.228	8 (7.3)	4 (7.3)	4 (7.3)	1.000	<0.001
Statins	109 (41.1)	22 (38.6)	87 (41.8)	0.774	0.066	45 (40.9)	22 (40.0)	23 (41.8)	1.000	0.037
Amiodarone	20 (7.5)	7 (12.3)	13 (6.2)	0.213	0.209	14 (12.7)	7 (12.7)	7 (12.7)	1.000	<0.001
Beta blockers	134 (50.6)	33 (57.9)	101 (48.6)	0.271	0.188	64 (58.2)	32 (58.2)	32 (58.2)	1.000	<0.001
ACEI or ARB	110 (41.5)	26 (45.6)	84 (40.4)	0.577	0.106	51 (46.4)	25 (45.5)	26 (47.3)	1.000	0.036
CCB	60 (22.6)	14 (24.6)	46 (22.1)	0.832	0.058	29 (26.4)	14 (25.5)	15 (27.3)	1.000	0.041
Digoxin	30 (11.3)	9 (15.8)	21 (10.1)	0.334	0.170	15 (13.6)	8 (14.5)	7 (12.7)	1.000	0.053

SMD, standardized mean difference; NA, not applicable; ACEI, angiotensin converting enzyme inhibitors; ARB, angiotensin receptor blocker; CCB, calcium channel blockers.

### Dynamic Changes of Anticoagulation Quality (TTR ≥60%) in AF Patients

**FIGURE 2:**
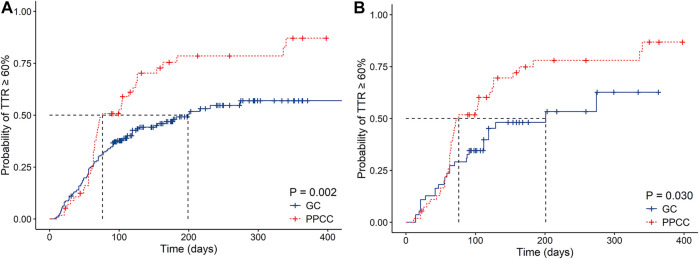
Kaplan–Meier estimates of cumulative percentages of patients with TTR ≥60% in **(A)** original cohort and **(B)** propensity score matching (PSM) cohort.TTR, time in therapeutic range; GC, general clinic; PPCC, physician–pharmacist collaborative clinic; Blue line represents the probability in the GC group and red line represents the probability in the PPCC group.

Considering the time when 50% of the patients achieved a TTR ≥60%, the PPCC group was demonstrated to have a shorter time than the GC group (76 vs. 199 days, *p* < 0.001), indicating that the PPCC model contributed to improved anticoagulation quality at the early stage of intervention (Figure 2A). During the mean follow-up of 203 days, more patients in the PPCC group were found to achieve a TTR ≥60% (73.7 vs. 47.1%, *p* = 0.002 by the log-rank test) than those in the GC group (Figure 2A). The results from the PSM cohort were in line with the primacy results (*p* = 0.03, log-rank test; Figure 2B).

### Predictors of High Anticoagulation Quality

A TTR ≥60% was considered indicative of high anticoagulation quality. In the univariate Cox regression analyses, treatment within a PPCC, stroke history, bleeding history, and the presence of more than four comorbidities were statistically associated with high anticoagulation quality (*p* < 0.1 for each variable). Multivariable analyses identified that treatment within a PPCC (HR: 1.84, 95% CI: 1.28–2.65, *p* = 0.001), stroke history (HR: 1.77, 95% CI: 1.11–2.83, *p* = 0.016), and bleeding history (HR: 10.04, 95% CI: 3.01–33.47, *p* < 0.001) were independent protective predictors associated with high anticoagulation quality, while the presence of more than four comorbidities (HR: 0.65, 95% CI: 0.46–0.92, *p* = 0.015) was an independent risk predictor related to high anticoagulation quality (Table 2). In the matched cohort, treatment within a PPCC was the only independent predictor related to high anticoagulation quality (HR: 1.73, 95% CI: 1.06–2.84, *p* = 0.03; Supplementary Table S1).

**TABLE 2:** Predictors associated with high anticoagulation quality (TTR>60%).

Variables	Crude analysis		Adjusted analysis	
	HR (95% CI)	*p* Value	HR (95% CI)	*p* Value
PPCC group	1.78 (1.24, 2.56)	0.002	1.84 (1.28, 2.65)	0.001
Age (years)	1.00 (0.98, 1.02)	0.801	—	—
Female	0.88 (0.63, 1.23)	0.466	—	—
Deep venous thrombosis	0.77 (0.11, 5.50)	0.792	—	—
Mechanical heart valve	0.89 (0.36, 2.18)	0.801	—	—
Valvular heart disease	0.98 (0.64, 1.52)	0.946	—	—
Coronary artery disease	0.81 (0.57, 1.14)	0.226	—	—
Hypertension	0.88 (0.63, 1.23)	0.453	—	—
Diabetes	0.77 (0.47, 1.26)	0.299	—	—
Heart failure	1.01 (0.69, 1.49)	0.963	—	—
History of stroke	1.56 (1.00, 2.45)	0.051	1.77 (1.11, 2.83)	0.016
History of bleeding	7.43 (2.29, 24.14)	0.001	10.04 (3.01, 33.47)	< 0.001
Myocardial infarction	1.09 (0.27, 4.39)	0.908	—	—
≥4 Comorbidities	0.75 (0.53, 1.04)	0.085	0.65 (0.46, 0.92)	0.015
Antiplatelet agents	0.98 (0.60, 1.62)	0.952	—	—
Statins	0.96 (0.69, 1.35)	0.831	—	—
Amiodarone	0.96 (0.50, 1.82)	0.890	—	—
Beta blockers	0.77 (0.55, 1.08)	0.129	—	—
ACEI or ARB	0.81 (0.57, 1.14)	0.221	—	—
CCB	1.03 (0.70, 1.54)	0.864	—	—
Digoxin	0.92 (0.55, 1.56)	0.768	—	—

HR, hazard ratio; CI, confidence interval; ACEI, angiotensin converting enzyme inhibitors; ARB, angiotensin receptor blocker; CCB, calcium channel blockers.

### Development of the Nomograms to Predict High Anticoagulation Quality

**FIGURE 3:**
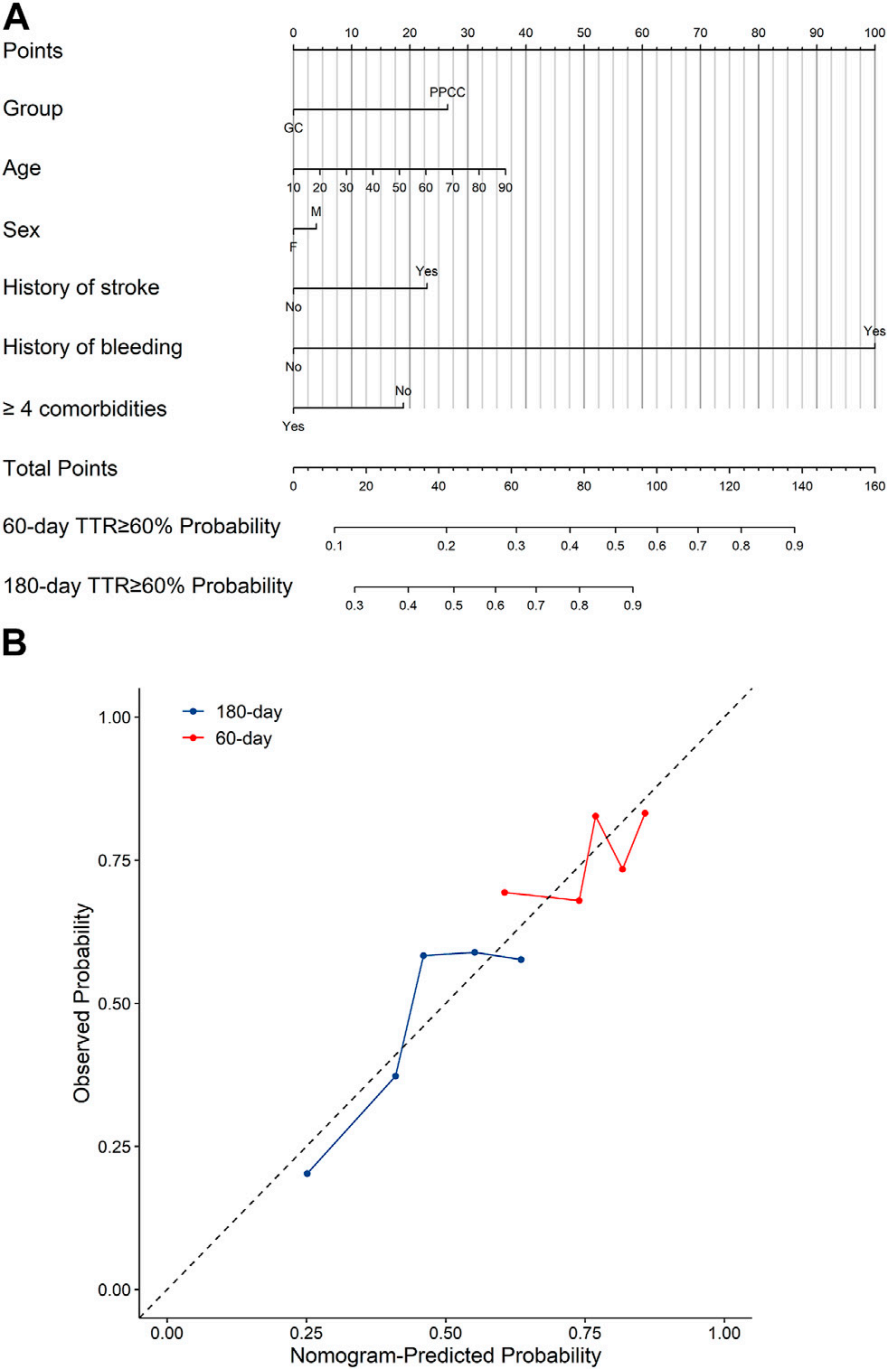
**(A)** The nomograms for predicting the probability of TTR ≥60% and **(B)** the calibration curves of the nomograms. GC, general clinic; PPCC, physician–pharmacist collaborative clinic; M, male; F, female; Blue line represents the probability of 180-days TTR ≥60% and red line represents the probability of 60-days TTR ≥60%. A smaller distance of the scatter points from the dotted line indicates better calibration.

The nomogram for predicting high anticoagulation quality was created based on demographics (age and sex), as well as the following four independent prognostic factors: treatment within a PPCC, stroke history, bleeding history, and the presence of more than four comorbidities (Figure 3A). Higher total points based on the sum of the assigned number of points for each factor in the nomogram were associated with a higher probability of TTR ≥60% within 60 and 180 days. For example, a 70-year-old female patient who had a history of stroke and received warfarin treatment within a PPCC would have a total of 77.5 points (27.5 points for age, 0 point for females, 23 points for stroke history, and 27 points for treatment within a PPCC) for a predicted 60-day and 180-day TTR ≥60% with probability of 42 and 79%, respectively (Supplementary Figure S1). The performance of the model was assessed by discrimination and calibration. The nomogram demonstrated a moderate predictive ability in predicting high anticoagulation quality, with an unadjusted c-index of 0.718 (95% CI 0.669–0.767) and a bias-corrected c-index of 0.718 (Figure 3B). In addition, another nomogram based on 208 patients in GC group achieved a similar predictive accuracy (c-index: 0.610, 95% CI: 0.650–0.770) (Supplementary Figure S2). All calibration plots graphically showed a moderate agreement between the TTR ≥60% probabilities predicted by the nomogram and the actual probabilities (Figure 3B; Supplementary Figure S2).

### Clinical Adverse Events Between the PPCC Group and GC Group

A comparison of clinical outcomes between the PPCC and GC groups is illustrated in Table 3. During a mean follow-up period of 203 days, thromboembolic and bleeding events occurred in 14 (5.3%) and 11 patients (4.1%), respectively. No significant differences were observed in the incidences of thromboembolic events (5.3 vs. 5.3%, *p* = 1.000) and bleeding events (3.5 vs. 4.3%, *p* = 1.000) between patients in the PPCC and GC groups. Thromboembolic events included 12 cases of ischemic stroke, one case of myocardial infarction, and one case of peripheral venous thrombosis. Three patients who experienced major bleeding events primarily had hematuria, and three patients had gastrointestinal bleeding. Meanwhile, a few patients experienced minor bleeding, such as epistaxis, subcutaneous hemorrhage, and subconjunctival hemorrhage.

**TABLE 3:** Comparison of clinical outcomes between PPCCs group and GCs group.

Outcomes	PPCCs (*n* = 57)	GCs (*n* = 208)	*p* Value
Thromboembolic events, *n* (%)	3 (5.3%)	11 (5.3%)	1.000
Ischaemic stroke, *n* (%)	3 (5.3%)	9 (4.3%)	0.725
Myocardial infarction, *n* (%)	0 (0.0%)	1 (0.5%)	1.000
Peripheral venous thrombosis, *n* (%)	0 (0.0%)	1 (0.5%)	1.000
Bleeding events, *n* (%)	2 (3.5%)	9 (4.3%)	1.000
Epistaxis, *n* (%)	1 (1.8%)	0 (0.0%)	1.000
Hemoptysis, *n* (%)	0 (0.0%)	1 (0.5%)	0.251
Haematuria, *n* (%)	0 (0.0%)	3 (1.4%)	1.000
Gastrointestinal haemorrhage, *n* (%)	0 (0.0%)	3 (1.4%)	1.000
Subcutaneous hemorrhage, *n* (%)	1 (1.8%)	1 (0.5%)	0.385
Subconjunctival hemorrhage, *n* (%)	0 (0.0%)	1 (0.5%)	0.251

## Discussion

### Major Findings and Interpretation

This study provides new insights into the role of PPCC in the care of AF patients receiving warfarin. The major findings were as follows: 1) the clinical characteristics of enrolled patients, including age and gender distribution, comorbidities, concomitant medications, were similar with the reported Chinese cohort of AF (Hu et al., 2019), indicating a good population representativeness; 2) the PPCC model could significantly improve the anticoagulation quality of warfarin, as indicated by shorter time and more patients to achieve a TTR ≥60% than the GC model in both original and matched cohorts; 3) multivariable analyses identified treatment within a PPCC, stroke history, and bleeding history as independent protective predictors, while the presence of more than four comorbidities as an independent risk predictor of high anticoagulation quality; 4) based on explored factors, a nomogram was created to predict anticoagulation quality, which can be used to inform the prognosis of patients, as well as to make individual decisions regarding anticoagulation treatment.

### Role of the Clinical Pharmacists in the Care of AF Patients Receiving Warfarin

High-quality anticoagulant therapy was the key to ensuring the efficacy and safety of warfarin administration in AF patients (Singer et al., 2013). The risk of death, bleeding, stroke, or other complications was associated with the quality of TTR control and was higher in patients with TTR <60% (White et al., 2007). As is well known, the administration of warfarin is a greater challenge in Chinese patients, who generally showed abnormal sensitivity to warfarin and had a higher risk of major bleeding than Caucasians (Yu et al., 1996; Li et al., 2015). In recent years, clinical pharmacists have gradually participated in the management of anticoagulants. Pharmacist-managed anticoagulation clinics have been established previously, and some success in medication education, dose adjustment, and regular follow-up monitoring have been achieved in certain countries, such as in the United States, New Zealand, and South Korea (Choe et al., 2002; Harrison et al., 2015; Rose et al., 2017). Compared with the above countries, the development of clinical pharmacies is relatively slow in China, where anticoagulation management is mainly the domain of physicians. The establishment of PPCCs at our institution is an attempt for pharmacists to take part in anticoagulant management. Based on the patients’ characteristics and medication status, the pharmacists in PPCCs assisted the physicians with the individualized medication regimen and provided detailed medication education, including diet, drug interactions, and INR monitoring for the patients. In the subsequent follow-up, the pharmacists adjusted the warfarin dose according to the instant INR and conducted medication education repeatedly for the patients. Previous studies have showed that the TTR level of warfarin users could be increased by 8.0–39.9% after pharmaceutical services (Motycka et al., 2012; Viquez-Jaikel et al., 2017; Marcatto et al., 2018). However, these studies only focused on the relationship between pharmacists’ intervention and outcomes at a certain time point. To our knowledge, this is the first study that estimated the influence of the continuous intervention of the PPCC model on the dynamic changes of TTRs in AF patients. The time to achieve a TTR ≥60% in the PPCC group was shorter than that in the GC group. In addition, approximately three-quarters of patients in the PPCC group achieved a target TTR ≥60%. Overall, the PPCC model at our institution could play an important role in improving the quality of warfarin therapy by assisting patients in drug management, as well as by strengthening medication education.

### Prediction Model of Anticoagulation Quality

Currently, the anticoagulation quality of warfarin in AF patients could be predicted by the SAMe-TT_2_R_2_ score (Apostolakis et al., 2013) or PROSPER score (Lin et al., 2017). The SAMe-TT_2_R_2_ score can be calculated for each individual by the following factors: female (1 point); age <60 years (1 point); medical history of >2 comorbidities (such as hypertension, diabetes, coronary artery disease/myocardial infarction, peripheral arterial disease, congestive heart failure, previous stroke, pulmonary disease, and hepatic or renal disease; one point); treatment with interacting drugs (e.g., amiodarone for rhythm control; one point); tobacco use (within 2 years; two points); and non-white (2 points), and a score of >2 was associated with suboptimal anticoagulation control. Given that the non-Caucasian race already confers two points in the SAMe-TT_2_R_2_ score and non-Caucasians accounted for only 9.8% of patients in the derivation cohort, the discrimination performance of the SAMe-TT_2_R_2_ score would be reduced in the Chinese AF population, with reduced sensitivity and negative predictive value after recalibration (Chan et al., 2016). In addition, this score was developed in 2013 when the awareness of standardized anticoagulant management was not strong enough with rare clinical pharmacists in China. In recent years, more pharmacists have gradually participated in anticoagulant management in various ways, which could make a difference to anticoagulation quality in China. Hence, this score may not be applicable to the current Chinese population. The PROSPER score can be calculated for each individual by the following factors: pneumonia (1 point); renal dysfunction (2 points); oozing blood (1 point); staying in hospital ≥7 days (1 point); pain medication use (1 point); no enhanced anticoagulation services (4 points); and Rx for antibiotics (1 point), and a score of >2 is predictive of having poor TTR. Of note, the factor of dedicated and structured anticoagulation management was incorporated into the score and was assigned the maximum score. Nevertheless, the PROSPER score was only dedicated to patients aged ≥65 years, which limited the extrapolation to large patient populations in real clinical settings. In addition, this score was mainly derived from the Caucasian population (91.3%) and has not been validated in Chinese populations to date. Accordingly, these individual factors that constitute the two scores may affect TTR differently across ethnic groups, with different lifestyles, values, and beliefs. Neither of the two scores took the time factor and the pharmacist factor into account. Our study found that treatment at one of the PPCCs and several clinical factors, such as stroke history, bleeding history, and more than four comorbidities, were related to anticoagulation quality. The role of PPCCs in the care of AF patients has been identified in the said analysis. History of stroke and bleeding as risk factors for poor INR control in the SAMe-TT_2_R_2_ score and PROSPER score, respectively, influenced anticoagulation quality in our study positively, which was consistent with results of some previous studies (Wypasek et al., 2016; Bjorck et al., 2019; Tiili et al., 2019). The possible reason is that AF patients who experienced embolic stroke or bleeding were more compliant to anticoagulant treatment than AF patients treated with prophylaxis only. Previous studies have also found that the coexistence of multiple diseases could significantly reduce the quality of INR control in AF patients receiving warfarin (Davis et al., 2005; Apostolakis et al., 2013), which can be explained by the increased number of hospitalizations required each year due to the comorbidities, which, in turn, led to poor anticoagulation quality in AF patients receiving warfarin.

Nomogram, as a graphical model calculating the probability of the outcome, has been improved as a feasible model in risk prediction and has been widely used in recent years (Lei et al., 2016; Serenari et al., 2020; Tian et al., 2020). To our knowledge, this study is the first to explore a feasible nomogram for predicting anticoagulation quality in Chinese AF patients receiving warfarin. In the nomogram of probability estimation, we combined the above independent predictors, as well as demographics (age and sex) that have been generally associated with the anticoagulant effect of warfarin (International Warfarin Pharmacogenetics et al., 2009; Bernaitis et al., 2016; Marcatto et al., 2016). Taken together, these predictors performed considerable property to predict anticoagulation quality in AF patients in the nomogram, with the moderate discriminatory ability and calibration. Considering the absence of clinical pharmacists in some hospitals, another nomogram with moderate discrimination performance after excluding the PPCC factor was also developed. These nomograms could help physicians and pharmacists to predict the anticoagulation quality of patients and to make individualized medication decisions or services for each patient. As an example, for patients predicted to have a low 60-day and 180-day TTR ≥60% probability, according to the relevant contributors to the score, pharmacists could provide them with individualized pharmaceutical care such as detailed drug management in those with multiple comorbidities or intensive medication education to improve the possibility of achieving high anticoagulation quality. We believe that the established nomogram represents a more precise prognostic model in the Chinese population, compared with previous scoring systems.

### Strengths and Limitations

The strengths of this study were as follows: first, to our knowledge, this is the first study to evaluate the role of the PPCC model on the dynamic changes of TTRs; second, this study established the first nomogram to predict high anticoagulation quality in Chinese AF patients receiving warfarin; third, PSM analysis was used to reduce the confounding factors. However, this study had some limitations. First, this was a retrospective, observational study that could introduce selection bias. Second, some of the possible determinants of TTR, such as diet information and patient genotypes (VKORC1 and CYP2C9), are not available in our study, which may limit the performance of the nomogram. Third, no difference in clinical adverse events was observed due to the limited follow-up time. Finally, given the limited number of samples in the single-center study, the prediction was not validated; thus, prospective studies with more samples and multicenter studies need to be carried out for internal and external validation.

## Conclusion

The PPCC model showed a faster positive effect on the improvement of anticoagulation quality in AF patients receiving warfarin. The nomogram created might be an effective tool to predict the anticoagulation quality and could aid physicians and pharmacists in the selection of patients who will likely benefit from sustained and active intervention.

## Data Availability

The raw data supporting the conclusion of this article will be made available by the authors, without undue reservation.
