# Supplementary material for: Physician–Pharmacist Collaborative Clinic Model to Improve Anticoagulation Quality in Atrial Fibrillation Patients Receiving Warfarin: An Analysis of Time in Therapeutic Range and a Nomogram Development
Source: Front Pharmacol. 2021 Jun 9;12:673302. doi: 10.3389/fphar.2021.673302 (PMC8220138; doi:10.3389/fphar.2021.673302)
Supplement: Supplementary file 1 [file DataSheet1.docx]

**Supplementary Table 1. Predictors associated with high anticoagulation quality in the matched cohort**

| **Variables** | **Crude HR**  **(95%CI)** | ***P* value** | **Adjusted HR**  **(95%CI)** | ***P* value** |
| --- | --- | --- | --- | --- |
| PPCC group | 1.73 (1.06,2.84) | 0.030 | 1.73 (1.06,2.84) | 0.030 |
| Age (years) | 1.03 (1.00,1.06) | 0.044 |  |  |
| Female | 0.95 (0.59,1.53) | 0.822 |  |  |
| Deep venous thrombosis | 0.76 (0.10,5.48) | 0.784 |  |  |
| Mechanical heart valve | 0.25 (0.03,1.81) | 0.170 |  |  |
| Valvular heart disease | 0.60 (0.33,1.11) | 0.102 |  |  |
| Coronary artery disease | 1.04 (0.62,1.75) | 0.879 |  |  |
| Hypertension | 1.09 (0.66,1.78) | 0.744 |  |  |
| Diabetes | 1.41 (0.77,2.59) | 0.264 |  |  |
| Heart failure | 0.97 (0.56,1.68) | 0.909 |  |  |
| History of stroke | 1.56 (0.71,3.43) | 0.266 |  |  |
| ≥ 4 Comorbidities | 0.73 (0.45,1.19) | 0.205 |  |  |
| Antiplatelet agents | 0.89 (0.35,2.23) | 0.801 |  |  |
| Statins | 1.03 (0.63,1.67) | 0.917 |  |  |
| Amiodarone | 1.23 (0.60,2.49) | 0.572 |  |  |
| Beta blockers | 0.68 (0.42,1.10) | 0.113 |  |  |
| ACEI or ARB | 0.70 (0.43,1.14) | 0.157 |  |  |
| CCB | 1.13 (0.67,1.91) | 0.654 |  |  |
| Digoxin | 0.91 (0.45,1.85) | 0.797 |  |  |

HR: Hazard Ratio; CI: confidence interval; ACEI: angiotensin converting enzyme inhibitors; ARB: angiotensin receptor blocker; CCB: calcium channel blockers.


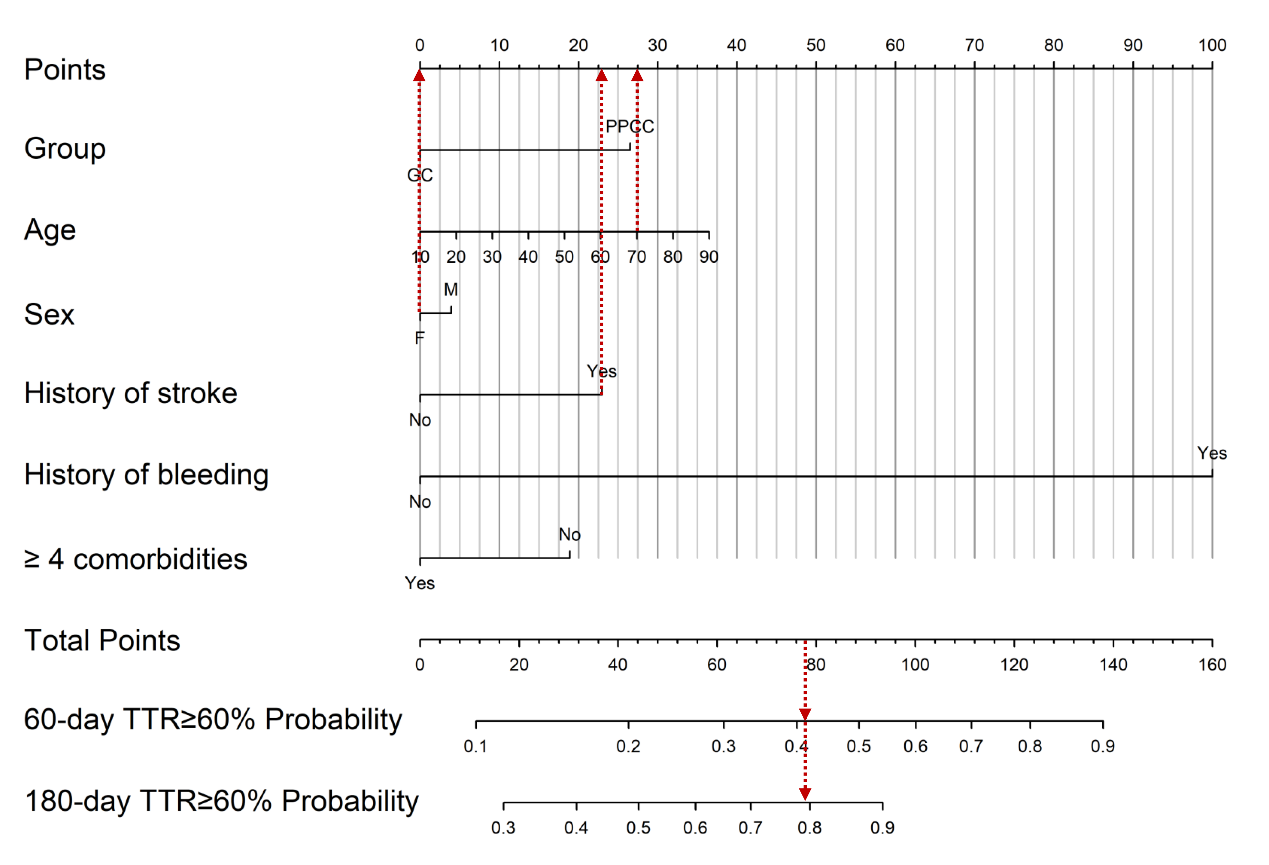


**Supplementary Figure 1. An example for nomogram practice.** For example, a 70-year-old female patient who had a history of stroke and received warfarin treatment within a PPCC would have a total of 77.5 points (27.5 points for age, 0 point for females, 23 points for stroke history, and 27 points for treatment within a PPCC) for a predicted 60-day and 180-day TTR ≥ 60% with probability of 42% and 79%, respectively.


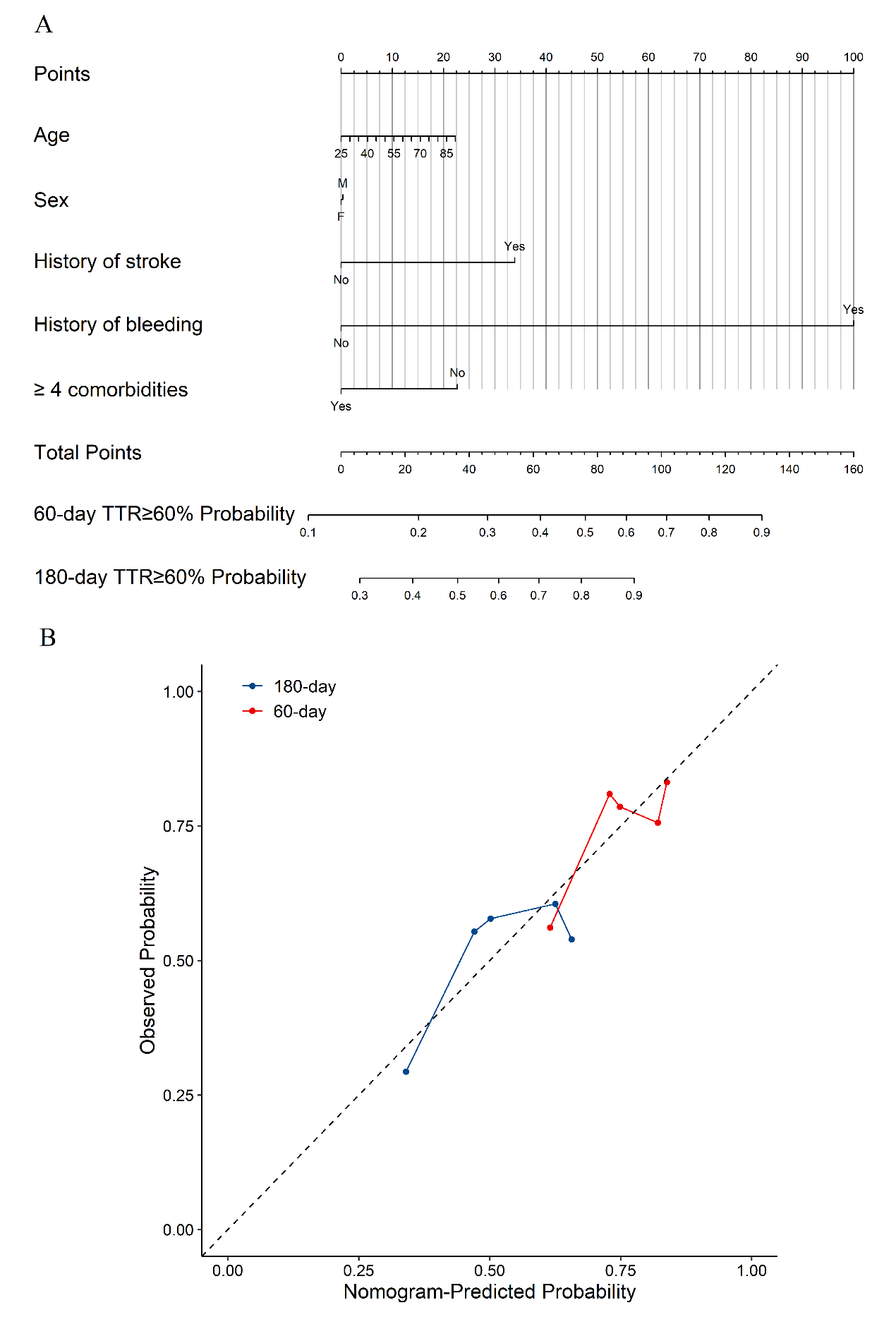


**Supplementary figure 2. The nomograms for predicting the probability of TTR ≥ 60% based on (A) patients in GC group; the calibration curves of the nomograms in (B) patients in GC group.** M: male; F: female; Blue line represents the probability of 180-days TTR ≥ 60% and red line represents the probability of 60-days TTR ≥ 60%. A smaller distance of the scatter points from the dotted line indicates better calibration.
